# Fusion of Multi-Spectral Features from UAVs to Improve the Accuracy of Total Soil Nitrogen Estimation During the Corn Growing Season

**DOI:** 10.3390/plants15203233

**Published:** 2026-10-21

**Authors:** Wenxing He, Minhua Yin, Yuhong Pu, Guoyun Shen, Yanxia Kang, Guangping Qi, Yayu Wang, Boda Li, Mingzhu Wang, Jiapeng Zhu

**Affiliations:** 1 College of Water Conservancy and Hydropower Engineering, Gansu Agricultural University, Lanzhou 730070, China; 1073325120999@st.gsau.edu.cn (W.H.); 1073325120974@st.gsau.edu.cn (Y.P.); qigp@gsau.edu.cn (G.Q.); wangyy@gsau.edu.cn (Y.W.); 1073324020375@st.gsau.edu.cn (B.L.); 1073324020379@st.gsau.edu.cn (M.W.); 1073324020383@st.gsau.edu.cn (J.Z.); 2 Gansu Provincial Center for Innovation in Agricultural Intelligent Water-Saving Technology, Lanzhou 730070, China; 3 Gansu Province Jingtai Chuan Power Irrigation Water Resource Utilization Center, Baiyin 730400, China; chosenone1107@163.com

**Keywords:** soil total nitrogen content, UAV multi-spectral, feature fusion, machine learning, maize

## Abstract

Rapid and accurate monitoring of soil total nitrogen (STN) is fundamental to optimising nitrogen fertiliser management, improving nitrogen use efficiency, and reducing agricultural non-point source pollution; however, traditional methods struggle to provide timely, large-scale, and continuous monitoring. Based on the relationship between maize canopy spectral characteristics and soil nitrogen distribution, this study acquired multi-spectral drone imagery during the jointing, tasselling, and grain filling stages, whilst simultaneously collecting soil samples from the 0–20, 20–40, and 40–60 cm soil layers. Using canopy reflectance (Band), vegetation indices (VIs), textural features (TF), and a fusion of these three features (Band+VIs+TF) as input variables, machine learning models, including Random Forest Regression (RFR), Gradient Boosted Decision Trees (GBDT), K-Nearest Neighbours (KNN), and XGBoost, were constructed to estimate STN. The results indicate that (1) STN is significantly correlated with spectral reflectance, vegetation indices, and textural features, with absolute correlation coefficients ranging from 0.60 to 0.89; (2) the accuracy of STN estimation by the four models showed a decreasing trend with increasing soil depth, with the highest accuracy observed in the 0–20 cm soil layer; (3) the validation accuracy of STN estimation followed the order: grain filling stage (R^2^ = 0.833) > tasselling stage (R^2^ = 0.794) > jointing stage (R^2^ = 0.761); (4) under feature fusion conditions, the STN estimation accuracy of all four models was superior to that of using a single feature, and the prediction accuracy of the XGBoost model (0.833) was higher than that of the other three models (0.701–0.734). In summary, during the grain filling stage of maize, using feature fusion as input variables and the XGBoost model can significantly improve the accuracy of STN estimation in maize fields, with the 0–20 cm soil layer yielding the best results.

## 1. Introduction

Soil total nitrogen (STN) is a key indicator of arable soil fertility and nitrogen supply capacity. Rapid, non-destructive assessment of the spatial distribution of STN in farmland is of great significance for precision nitrogen application, reducing excessive fertiliser use, controlling agricultural non-point source pollution, and maintaining sustainable soil productivity [1]. Traditional methods for determining soil total nitrogen rely on field sampling and laboratory chemical analysis; whilst these offer high precision, they are costly and time-consuming, making it difficult to meet the requirements for continuous monitoring and dynamic management at the field plot scale. Precision agriculture requires an understanding of the spatial variability of soil properties in the field, enabling zoned management and differentiated fertilisation based on this information [2]. Furthermore, optimising fertilisation practices alone is insufficient to dynamically match nitrogen requirements at different stages of crop growth; it is therefore necessary to integrate remote sensing monitoring technologies to enhance the timeliness and accuracy of nitrogen management in farmland [3].

Multi-spectral remote sensing using unmanned aerial vehicles (UAVs) offers advantages such as high spatial resolution, flexible data acquisition, and relatively low cost, and has been widely applied in crop nutrient diagnosis and soil property estimation [4,5]. Compared with satellite and manned aerial remote sensing, UAVs can mitigate the constraints imposed by cloud cover and revisit intervals, whilst acquiring high-resolution canopy spectral information at the field plot scale, thereby providing a vital data source for nutrient monitoring in agricultural fields [6,7,8,9]. However, multi-spectral imagery is susceptible to factors such as lens distortion, vignetting, and radiometric differences; consequently, standardised image pre-processing is a crucial foundation for ensuring the accuracy of inversion [10]. Previous studies have shown that soil nitrogen availability can indirectly alter the spectral response of the crop canopy by influencing leaf chlorophyll content, canopy structure, and photosynthetic processes, resulting in a certain degree of sensitivity to nitrogen changes in the visible and near-infrared bands [11,12]. However, single-band reflectance is susceptible to interference from soil background, canopy cover, and environmental conditions. Vegetation indices, which combine multiple bands to enhance nitrogen-related spectral information, such as NDVI, RVI and GI, have been used to estimate soil or crop nitrogen [13]; however, the stability of individual spectral features or vegetation index models across different growth stages remains limited [7].

In addition to spectral information, texture features extracted from greyscale co-occurrence matrices can describe the spatial structure and heterogeneity of the canopy, providing supplementary information for characterising differences in crop growth [14,15]. Previous studies have shown that fusing texture information with vegetation indices or canopy spectral features can mitigate the limitations of individual features and improve the accuracy of estimating nitrogen and related growth parameters [16,17]. The combined application of multi-temporal features and data from different sources also helps to improve the stability of crop growth parameter inversion [18]. At the same time, machine learning methods such as Random Forest, Gradient Boosting Trees, K-nearest Neighbours and XGBoost are capable of handling the complex non-linear relationships between spectral data, vegetation indices, and textural features, and have demonstrated good application potential in the estimation of various crop parameters [19]. However, existing studies have primarily focused on crop canopy nitrogen, biomass, or soil nutrients in a single soil profile; systematic research on the relationship between STN and multi-source canopy features at different growth stages and soil depths in maize grown in drought-prone irrigated areas remains scarce. In particular, there is a lack of comprehensive comparisons of the performance of different machine learning models under conditions where spectral, vegetation index, and textural features are fused.

The Jingtai-Chuan Pumped Irrigation District in Gansu Province is a typical continental arid agricultural region characterised by low precipitation and high evaporation; agricultural production is heavily reliant on pumped irrigation [20], whilst high inputs of chemical fertilisers increase the risk of nitrogen loss, soil fertility degradation, and non-point source pollution in farmland [1]. This study took maize as the subject of investigation, acquiring multi-spectral drone imagery during the jointing, tasselling, and grain filling stages, whilst simultaneously measuring soil total nitrogen (STN) in the 0–60 cm soil layer and extracting canopy reflectance, vegetation indices, and textural features. Based on the statistical relationship between canopy spectral response and soil nitrogen changes, Pearson correlation analysis was employed to screen for significant feature variables, and RFR, GBDT, KNN, and XGBoost regression models were constructed. The study systematically compared the performance of STN estimation across different growth stages, soil depths, and feature combinations. Specific objectives included (1) clarifying the response relationship between STN and spectral and textural features, as well as their variation characteristics with respect to growth stage and soil depth; (2) evaluating the impact of multi-source feature fusion on the accuracy of STN estimation; (3) comparing the performance differences and applicability of various machine learning models in STN estimation. This study provides an exploratory assessment of feature fusion and machine learning for STN estimation at a single site during one growing season. Given the limited number of experimental plots, the findings require independent validation before being extended to other fields, years, or regions.

## 2. Results and Analysis

### 2.1. Statistical Analysis of STN

**Figure 1:**
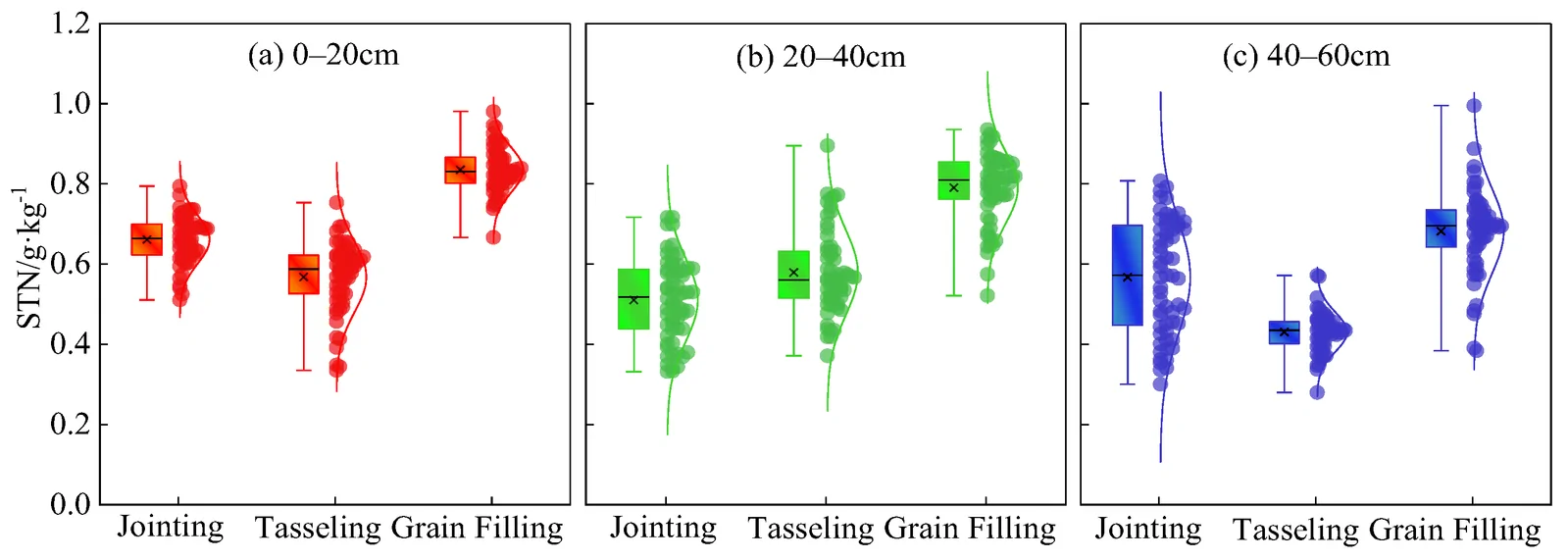
Soil total nitrogen (STN) content at different soil depths and growth stages. Note: Red, green, and blue represent the 0–20 cm, 20–40 cm, and 40–60 cm soil layers, respectively.

Statistical analysis (Table 1 and Figure 1) shows that STN in different soil layers across the three growth stages exhibited a certain degree of spatiotemporal variation. The standard deviation for each treatment combination ranged from 0.055 to 0.143 g·kg^−1^, with coefficients of variation ranging from 6.65% to 25.26%; overall, the variation was low to moderate. The mean STN values in the 0–20 cm and 40–60 cm soil layers followed a pattern of first decreasing and then increasing as the growing season progressed, whilst in the 20–40 cm soil layer, the value rose gradually from 0.506 g·kg^−1^ to 0.780 g·kg^−1^, indicating that the changes across different soil layers were not entirely consistent.

### 2.2. Correlation Analysis of STN

#### 2.2.1. Correlation Between Spectral Reflectance and STN

**Figure 2:**
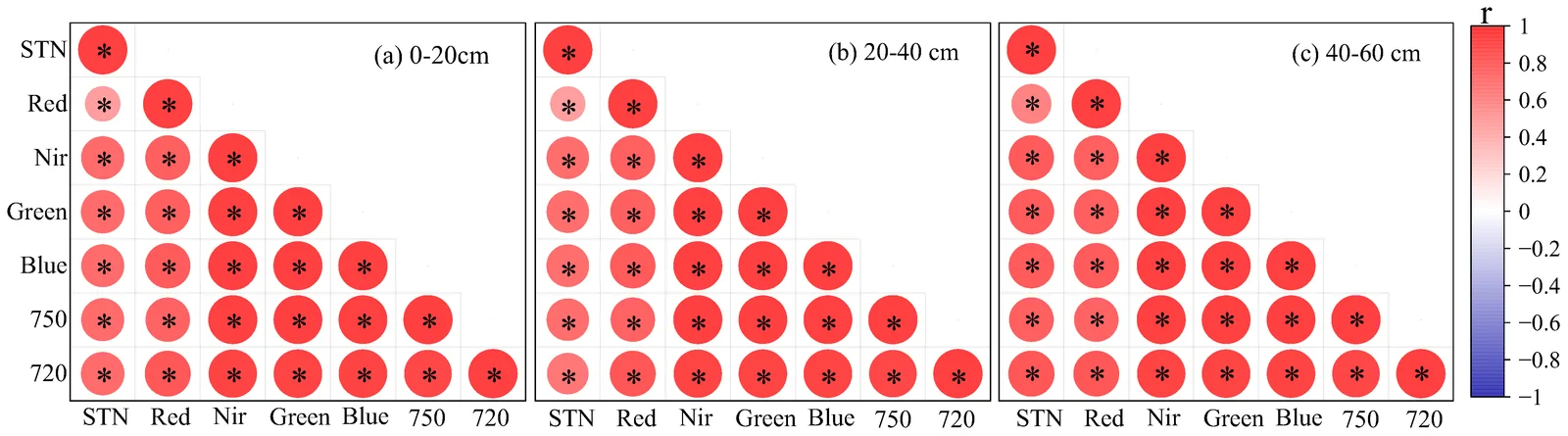
Correlation between STN and reflectance at the jointing stage. Note: * indicates *p* ≤ 0.05.

**Figure 3:**
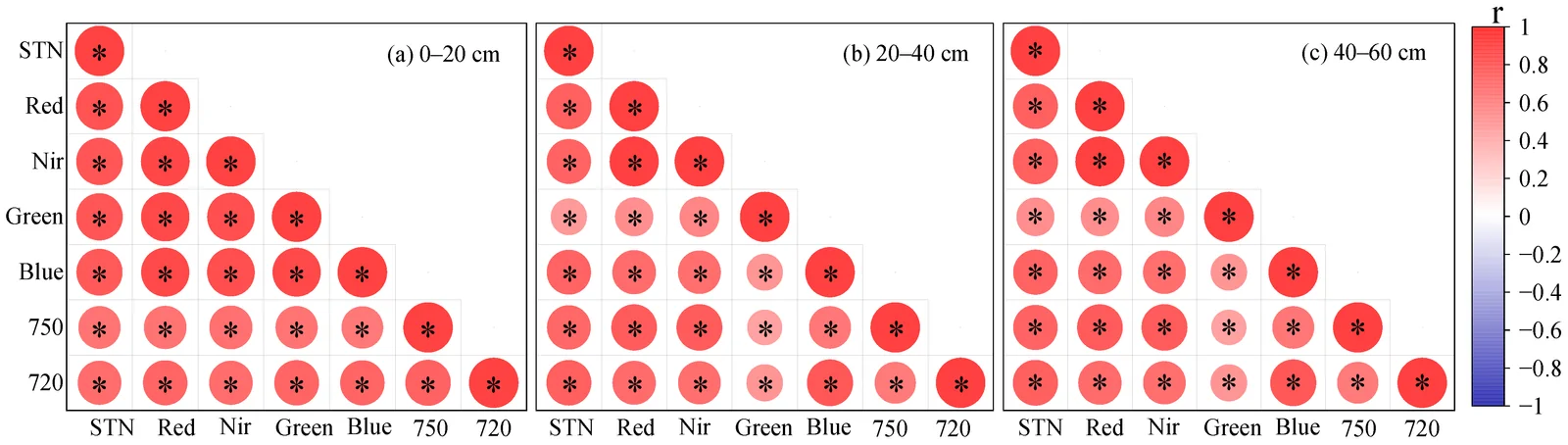
Correlation between STN and reflectance at the tasseling stage. Note: * indicates *p* ≤ 0.05.

**Figure 4:**
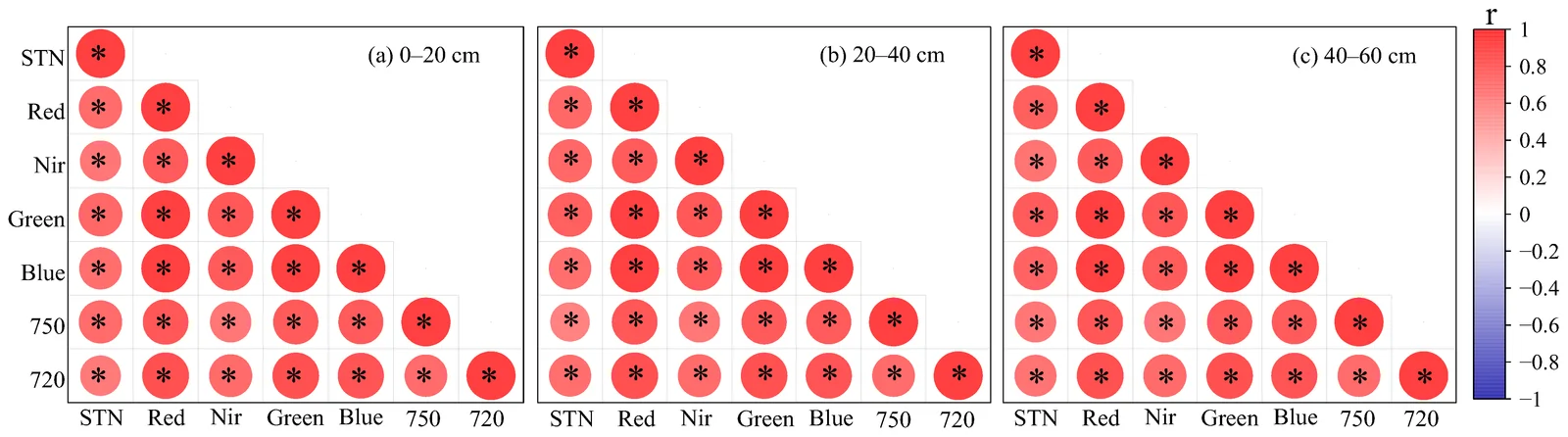
Correlation between STN and reflectance at the grain filling stage. Note: * indicates *p* ≤ 0.05.

Pearson correlation analysis between STN and spectral reflectance (Figure 2, Figure 3 and Figure 4) showed that the relationships varied with growth stage and soil depth. During the jointing stage, the bands that significantly correlated with STN across the soil layers were mainly NIR, Green, Blue, and Rededge 2 (750 nm), with |r| ranging from 0.748 to 0.845. During the tasselling stage, the correlation coefficients for the 0–20 cm soil layer all exceeded 0.87. With increasing soil depth, the significantly correlated bands were mainly Red, NIR, Blue, and Rededge 2 (750 nm), with |r| ranging from 0.783 to 0.838. During the grain filling stage, the significantly correlated bands across the soil layers were mainly Red, Green, Blue, and NIR (|r| ≥ 0.75). Overall, STN was more strongly correlated with spectral reflectance in the 0–20 cm soil layer than in the deeper layers.

#### 2.2.2. Correlation Between Vegetation Indices and STN

**Figure 5:**
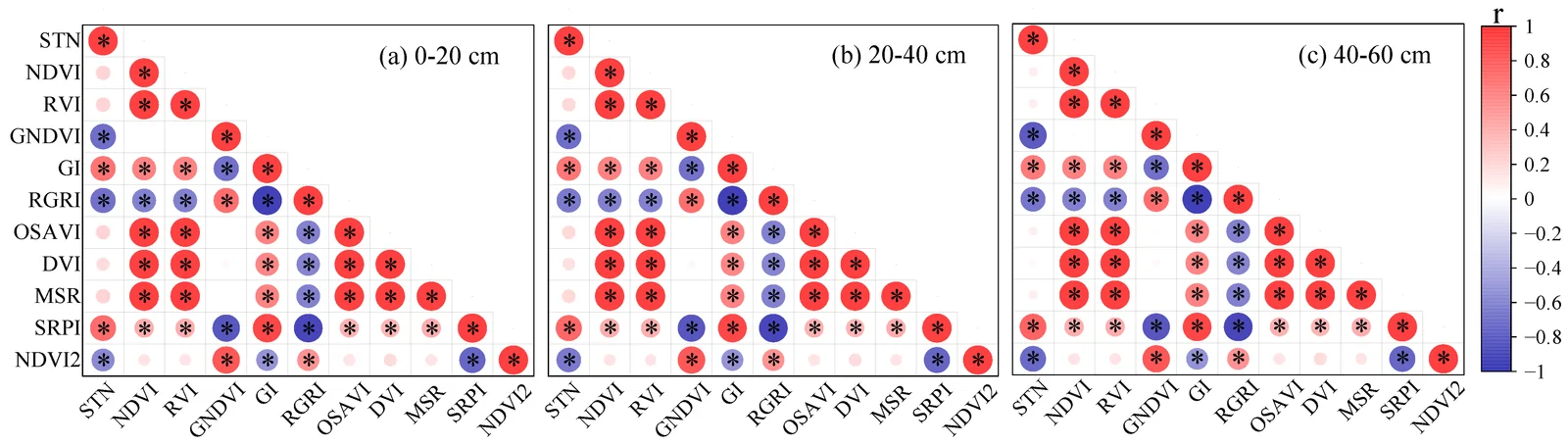
Correlation between STN and vegetation indices at the jointing stage. Note: * indicates *p* ≤ 0.05.

**Figure 6:**
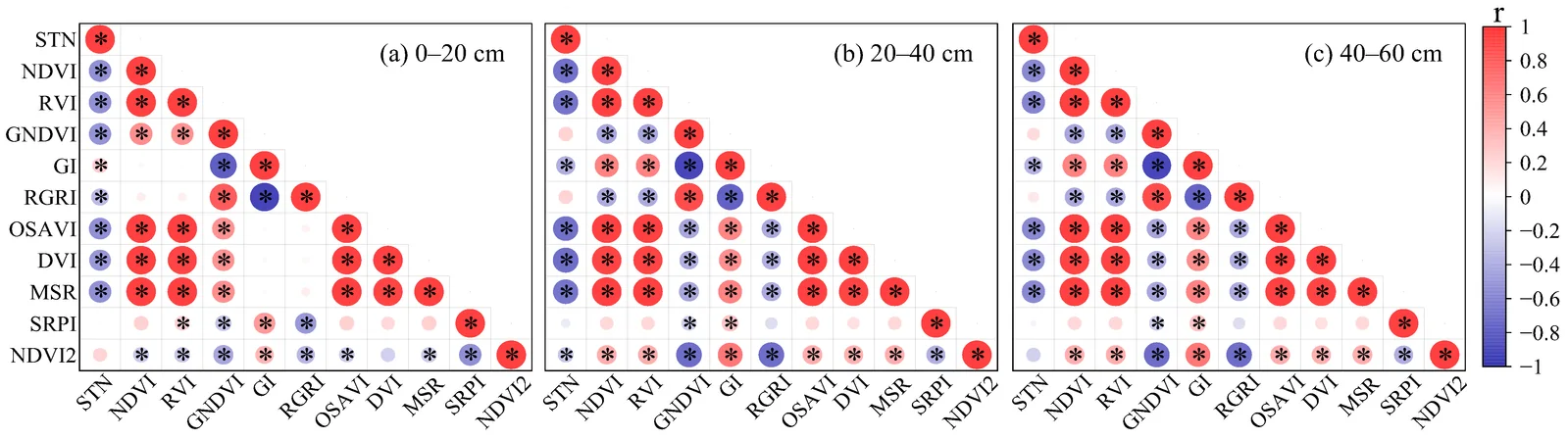
Correlation between STN and vegetation indices at the tasseling stage. Note: * indicates *p* ≤ 0.05.

**Figure 7:**
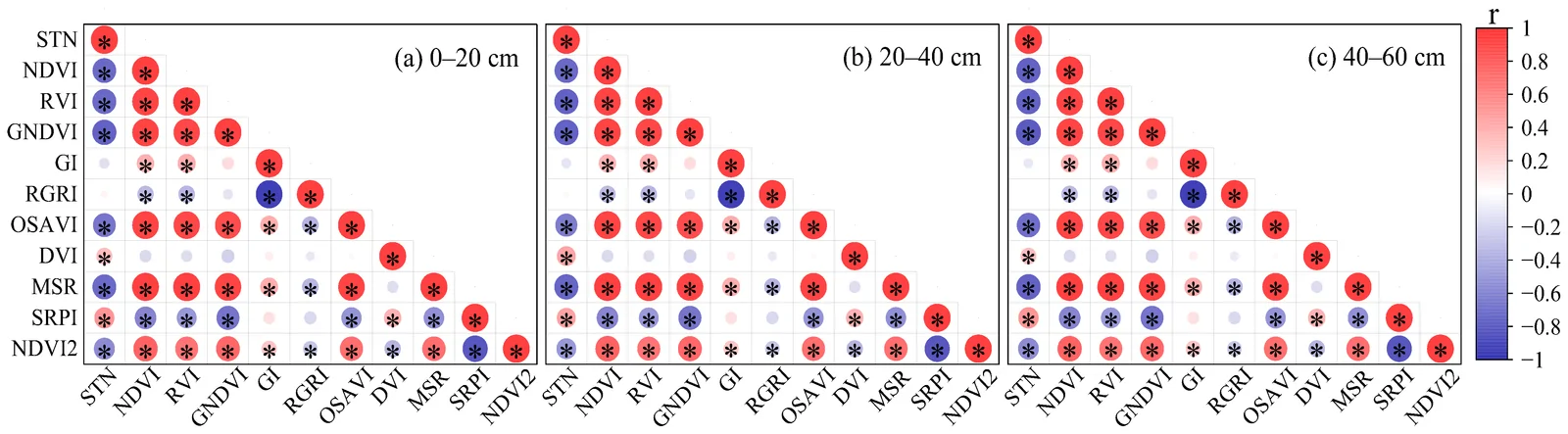
Correlation between STN and vegetation indices at the grain filling stage. Note: * indicates *p* ≤ 0.05.

Analysis of the Pearson correlation between STN and vegetation indices (Figure 5, Figure 6 and Figure 7) shows that, during the jointing stage, the indices significantly correlated with each soil layer were primarily GNDVI, GI, RGRI, and SRPI, with |r| ranging from 0.696 to 0.811. During the tasselling stage, the correlation coefficients varied with soil depth, with NDVI, RVI, and MSR being the primary indices in the 0–20 cm range (|r| > 0.57), in the 20–40 cm range, the predominant indices were NDVI, RVI, and OSAVI (|r| ≥ 0.717), and in the 40–60 cm range, the predominant indices were NDVI, RVI, and MSR (|r| ranging from 0.615 to 0.620). During the grain filling stage, NDVI, RVI, and GNDVI were the predominant indices across all soil layers, with |r| reaching 0.768–0.809 or higher. In summary, the correlation between vegetation indices and STN strengthened as the growth stage progressed; the correlation coefficients for all soil layers during the grain filling stage were higher than those during the jointing stage and the tasselling stage.

#### 2.2.3. Correlation Between Texture Features and STN

**Figure 8:**
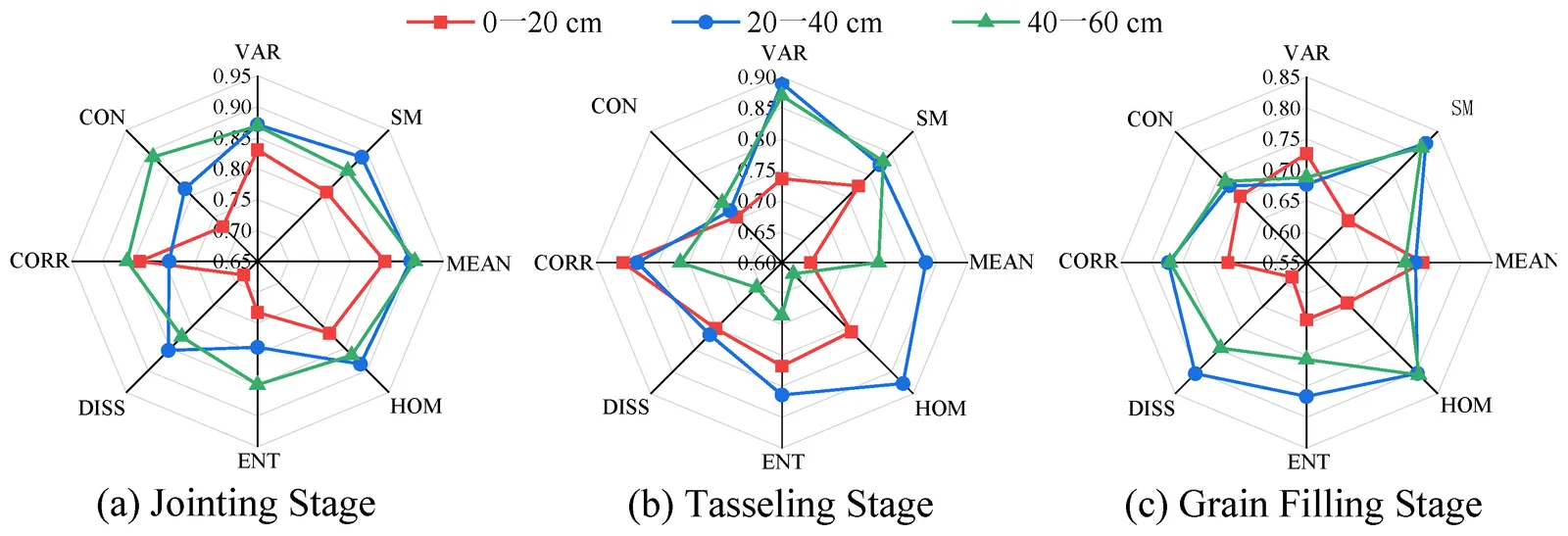
Correlation analysis between STN and texture features at different growth stages.

This study extracted eight types of texture features based on the Green band (MEAN, VAR, HOM, DISS, CON, ENT, SM, CORR) and conducted a correlation analysis with STN (Figure 8).

During the jointing stage, the 0–20 cm range was dominated by VAR, SM, and CON (|r| = 0.819–0.866); the 20–40 cm range was dominated by SM, MEAN, and HOM (|r| = 0.605–0.633); and the 40–60 cm range was dominated by VAR, MEAN, and HOM (|r| = 0.789–0.862). During the 0–20 cm stage of the tasselling period, SM, DISS, and CORR were predominant (|r| > 0.8); during the 20–40 cm stage, MEAN, ENT, and CORR were predominant (|r| = 0.832–0.857); and during the 40–60 cm stage, VAR, SM, and HOM were predominant (|r| = 0.732–0.862). During the grain filling stage, at 0–20 cm, SM, MEAN, and ENT were predominant (|r| = 0.719–0.801); at 20–40 cm, SM, HOM, and CORR were predominant (|r| = 0.778–0.847); and at 40–60 cm, SM, MEAN, and HOM were predominant (|r| = 0.718–0.851). In summary, the correlation between textural characteristics and STN varies with growth stage and soil depth, with the best performance generally observed during the grain filling stage.

### 2.3. Comprehensive Evaluation of Models

**Figure 9:**
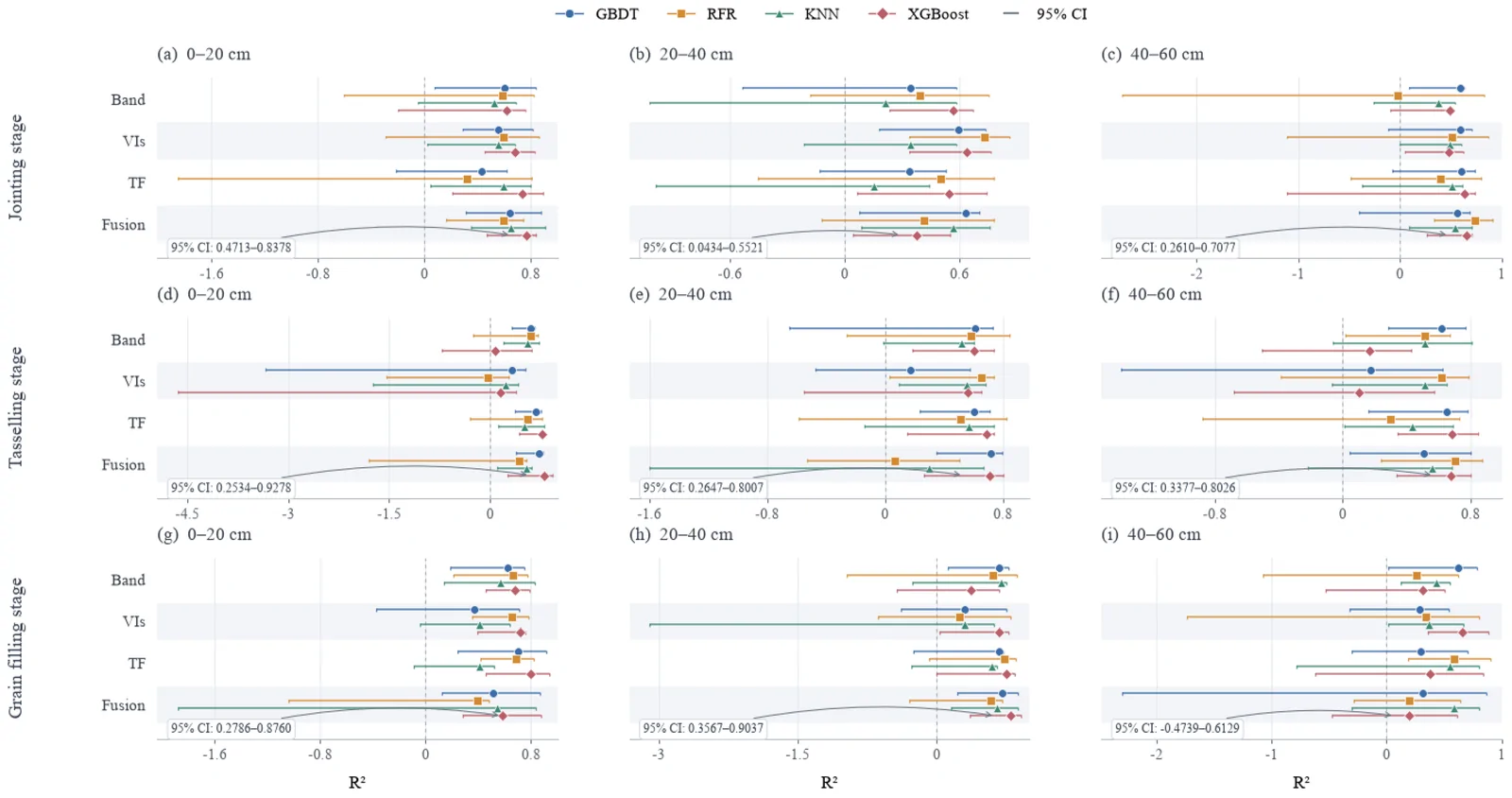
R^2^ values for the validation sets of each model across different growth stages and soil depths. Note: (**a**–**c**) represent the jointing stage, (**d**–**f**) the tasselling stage, and (**g**–**i**) the grain filling stage.

**Figure 10:**
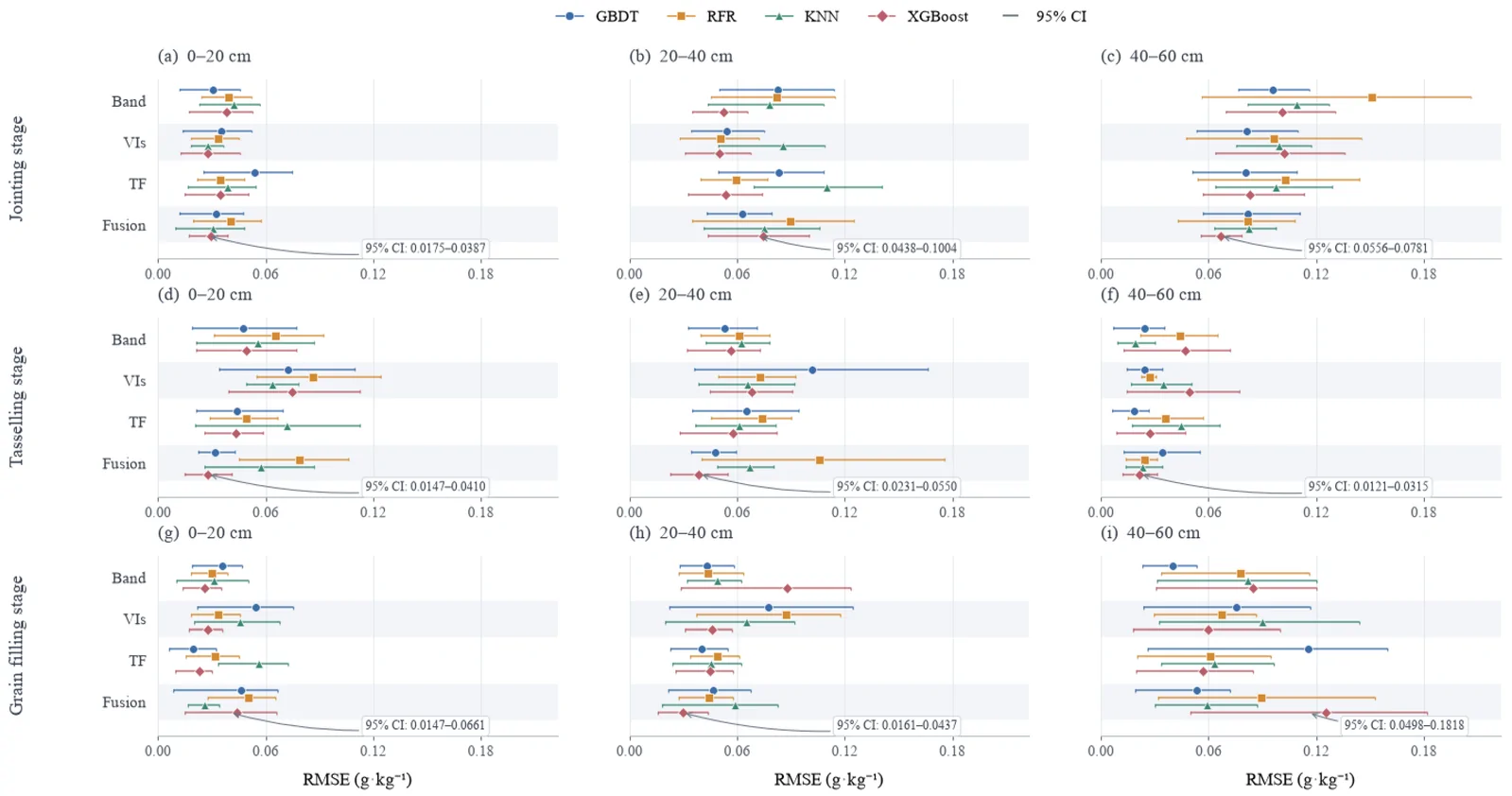
RMSE for each model across validation sets at different growth stages and soil depths. Note: (**a**–**c**) represent the jointing stage, (**d**–**f**) the tasselling stage, and (**g**–**i**) the grain filling stage.

**Figure 11:**
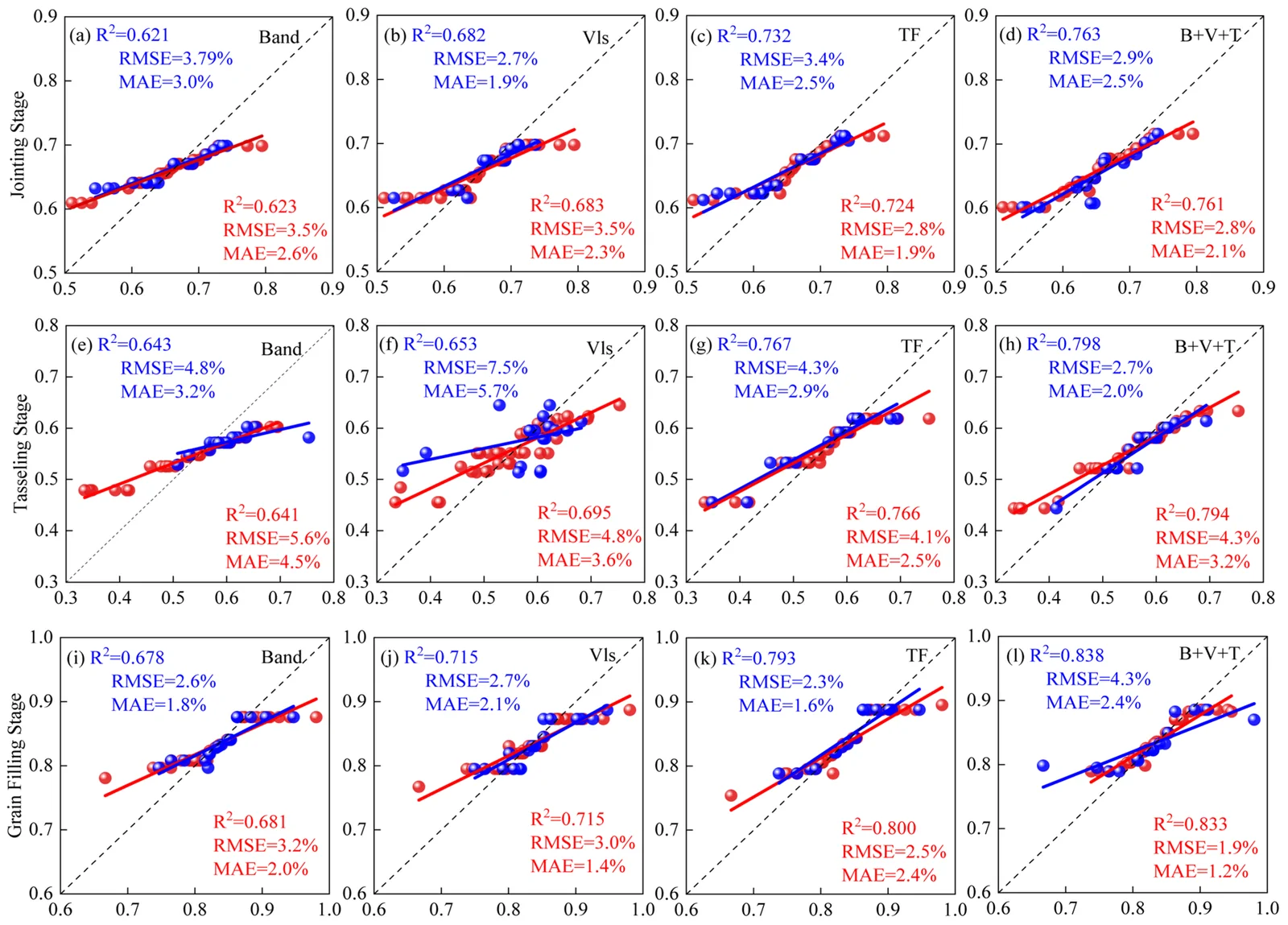
Validation and training sets of the optimal STN prediction model (XGBoost). Note: Blue represents the training set, and red represents the validation set. The RMSE and MAE in the figure are normalised by the mean of the measured values and are expressed as rRMSE (%) and rMAE (%), respectively. (**a**) Band at the jointing stage; (**b**) VIs at the jointing stage; (**c**) TF at the jointing stage; (**d**) B+V+T at the jointing stage; (**e**) Band at the tasselling stage; (**f**) VIs at the tasselling stage; (**g**) TF at the tasselling stage; (**h**) B+V+T at the tasselling stage; (**i**) Band at the grain filling stage; (**j**) VIs at the grain filling stage; (**k**) TF at the grain filling stage; (**l**) B+V+T at the grain filling stage.

For each growth stage and soil depth, the 60 samples from 10 experimental plots were randomly divided into a training set (70%; *n* = 42) and a validation set (30%; *n* = 18). Samples were randomly split at the sample level, with samples from the same plot included in both sets. Within-plot spatial dependence may therefore have inflated performance estimates, limiting conclusions about generalisation to unseen plots. Model parameter optimisation was carried out solely on the training set, whilst the validation set was subsequently used to assess the model’s generalisation ability. The STN prediction accuracy of four models—Random Forest Regression (RFR), XGBoost, Gradient Boosted Decision Trees (GBDT) and K-Nearest Neighbours (KNN)—was compared across different growth stages, soil depths, and input variables. Figure 9 and Figure 10 present the validation-set R^2^ and RMSE, respectively, for each model across these conditions. Figure 11 additionally reports the training and validation performance of the optimal XGBoost model, including MAE. These metrics represent point estimates of model performance under the training–validation split used in this study. XGBoost performed best across all growth stages and soil layers. In the 0–20 cm soil layer, when using the Band+VIs+TF fusion input, the validation set R^2^ values for XGBoost during the jointing stage, tasselling stage, and grain filling stage were 0.761, 0.794, and 0.833, respectively, with an average R^2^ of 0.796, all of which were higher than those achieved with the corresponding single Band, VIs, or TF inputs. The highest validation set R^2^ was obtained in the 0–20 cm soil layer during the grain filling stage. In summary, XGBoost demonstrated the strongest generalisation ability, and the fusion of multi-source features significantly improved estimation accuracy; the 0–20 cm soil layer during the grain filling stage was the best-performing monitoring combination under the conditions of this study.

### 2.4. Spatial Mapping of STN

**Figure 12:**
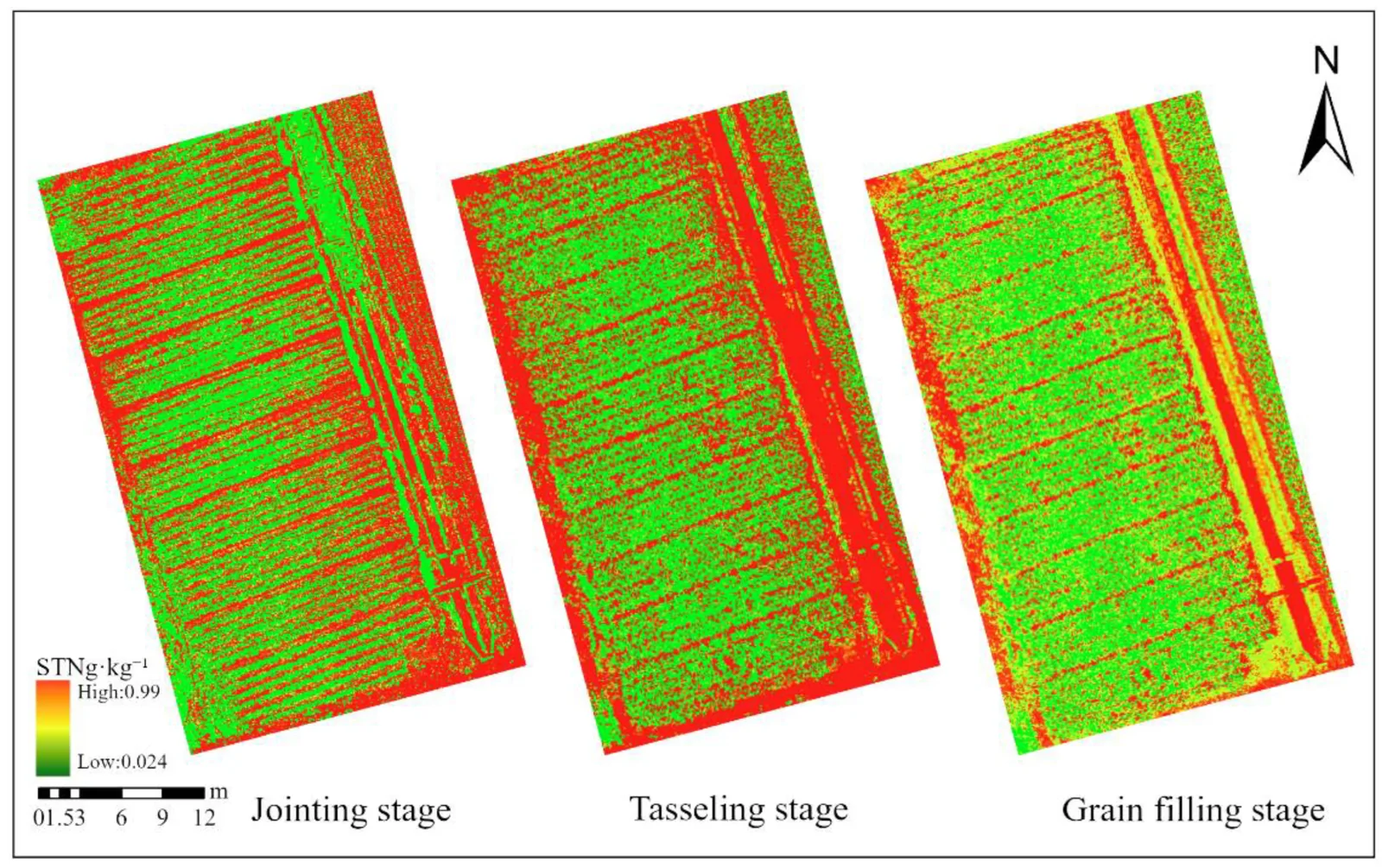
Spatial distribution of STN. Note: Spatial distribution of estimated STN in the 0–20 cm soil layer during the jointing, tasselling and grain filling stages. All three maps use the same colour scale, ranging from 0.024 to 0.99 g·kg^−1^.

In this study, the XGBoost model, which utilised a combination of Band, VIs, and TF as input features, achieved the highest estimation accuracy, ultimately yielding spatial distribution maps of STN for maize at various growth stages (Figure 12). The spatial estimation results indicate that there are significant differences in the spatial distribution range and areas of high and low values of STN across different growth stages. The total nitrogen content ranged from 0.39–0.73 g·kg to 1 during the jointing stage, 0.48–0.72 g·kg to 1 during the tasselling stage, and 0.68–0.85 g·kg to 1 during the grain filling stage. In terms of spatial distribution, high-nitrogen areas during the grain filling stage were mainly concentrated in the central and southern parts of the experimental field, whilst low-nitrogen areas were distributed along the periphery; this may be related to the uniformity of fertiliser application and microtopography.

## 3. Discussion

### 3.1. Effects of Soil Depth on STN Estimation Accuracy

The accuracy of total soil nitrogen estimation generally decreases with increasing soil depth; the R^2^ value for the validation set in the 0–20 cm soil layer was consistently higher than that for the 20–40 cm and 40–60 cm soil layers across all growth stages and models, and this pattern was largely unaffected by the combination of input variables or the type of model. The higher prediction accuracy for the 0–20 cm soil layer may partly relate to the concentration of maize roots in topsoil reported in previous studies [21]. Nitrogen leaching and accumulation in deeper layers [22] may also contribute to differences in the relationship between STN and canopy characteristics. However, biomass, root distribution, and nitrogen uptake were not measured in this study; these mechanisms should therefore be considered possible explanations rather than demonstrated causes.

Similar variations in estimation accuracy are also observed in remote sensing inversions of soil properties such as organic carbon, moisture, and salinity; the accuracy of UAV multi-spectral inversions of soil salinity at different depths shows marked differences, but relevant studies have not yet considered the influence of different growth stages under crop cover conditions [23]. Furthermore, under bare-soil conditions, the R^2^ value for the RF-FDR validation set in the 0–20 cm surface STN model—constructed using UAV hyperspectral data (398–1003 nm) and PLSR, RR, and RF methods—reached 0.859 [11]. Compared with such studies on bare soil, single soil layers, and hyperspectral data, this study focuses on multiple growth stages of maize, multiple soil layers, and multiple spectral conditions, with a core emphasis on the comparison of Band+VIs+TF fusion and machine learning models. Bare-soil spectra reflect surface soil properties, whereas UAV imagery under maize canopy cover primarily represents canopy characteristics and cannot directly detect nitrogen in the 20–40 cm and 40–60 cm layers. Deep-layer STN is therefore estimated through statistical associations that may vary with growth stage, canopy cover, and soil depth. Root uptake and canopy responses may provide a physiological link, but this mechanism was not verified in this study. Nevertheless, the R^2^ value for the 0–20 cm fusion XGBoost validation set during the grain filling stage still reached 0.833. It should be noted that this study was based on field observations from a single year only. Interannual fluctuations in precipitation and changes in irrigation regimes may alter vertical nitrogen migration and its coupling with canopy spectroscopy; consequently, the long-term stability of these hierarchical patterns still requires verification through multi-year continuous experiments, combined with multi-year time-series remote sensing and soil sampling data to further enhance the estimation capability of deep-layer STN.

### 3.2. Effects of Multi-Source Feature Fusion on STN Estimation Accuracy

This study established four sets of input variables: canopy reflectance (Band), vegetation indices (VIs), textural features (TF), and a fusion of the three (Band+VIs+TF). The results indicate that all three types of individual features are significantly correlated with STN and can serve as effective estimation variables; however, there are differences in their estimation capabilities and applicability. Following the fusion of multi-source features, model accuracy was systematically improved. Notably, XGBoost outperformed RFR, GBDT, and KNN under all four input conditions. When using the ‘Band+VIs+TF’ combination, XGBoost achieved an R^2^ value exceeding 0.76 on the validation set for the 0–20 cm soil layer across all three growth stages, representing average improvements of 23.1 per cent, 16.9 per cent, and 4.65 per cent compared with the use of Band, VIs, and TF individually. Previous studies have similarly demonstrated that the fusion of spectral and textural information can improve the accuracy of nitrogen estimation [16]. This study further extends this finding to multiple growth stages of maize and stratified soil conditions in arid regions.

The correlation between single-band reflectance and STN is easily influenced by the growth stage, canopy cover, and soil depth, with particularly significant fluctuations observed during periods of low canopy cover and in deep soil layers. Previous studies have indicated that, whilst raw band reflectance can directly reflect spectral changes, it is susceptible to interference from non-target factors such as soil background and lighting conditions, and is relatively unstable [11]. Vegetation indices (VIs) generally exhibit higher estimation accuracy than single-band measurements; however, during the period from tasselling to grain filling stage, when canopy cover is high, the improvement in accuracy tends to be limited, exhibiting a certain degree of saturation. This is consistent with findings that, under high-cover conditions, the ability of vegetation indices to distinguish nitrogen differences declines [16]. In contrast, texture features (TF) can supplement information on canopy spatial structure that is difficult to characterise using spectral features alone. Relevant studies have shown that the introduction of texture features can significantly improve the accuracy of nitrogen estimation [24], with an increase in model R^2^ ranging from 9.77% to 15.54% [25]; furthermore, indices combining spectral and texture data perform better than single indices [26], indicating that texture information has a stable synergistic effect.

The optimal models identified in different studies are not entirely consistent. In previous research utilising hyperspectral data from unmanned aerial vehicles (UAVs) to estimate soil NO_3_^−^–N at depths of 0–50 cm, the model R^2^ increased from 0.53 to over 0.88 following the inclusion of fertilisation gradient labels, with PLSR performing slightly better than XGBoost [27]. That study centred on hyperspectral data, NO_3_^−^–N, and prior information on fertilisation gradients; as the systematic differences were primarily driven by fertilisation gradients, PLSR was better suited to capturing the main trends. In contrast, the present study focuses on maize STN in arid irrigated areas, where differences between fertilisation treatments are relatively small. The canopy–soil relationship is jointly influenced by growth stage and soil depth, and the interactions between non-linear and multi-source features are more pronounced; consequently, XGBoost performs better under these integrated conditions.

Band, VIs, and TF characterise canopy responses to changes in STN from three dimensions: spectral response, biochemical status, and spatial structure, respectively, and the information they provide is highly complementary, which is consistent with the theory of multidimensional ‘spectral fusion’ [28]. Previous studies have also shown that the fusion of spectral indices and texture indices can reduce soil background interference and enhance canopy nitrogen responses [16]. This study further found that this fusion advantage persists across different growth stages and soil depths, being particularly pronounced in deep soil layers, under low canopy cover during the jointing stage, and under high canopy cover during the grain filling stage. This may be attributed to the varying contributions of different features across growth stages: during the jointing stage, spectral and textural information can compensate for the instability of spectral indices (VIs); during the tasselling stage, VIs respond more strongly to biochemical changes in the canopy; whilst during the grain filling stage, textural information can compensate for information loss caused by spectral saturation under high-canopy-cover conditions. These results suggest that model performance should be evaluated separately for different growth stages, with canopy cover and spectral saturation considered possible contributing factors.

Under the random training–validation split used in this study, XGBoost combined with Band+VIs+TF achieved higher STN estimation accuracy than the corresponding individual feature groups. This finding suggests the potential of feature fusion for STN estimation within the present experimental dataset. However, these results do not establish the model’s predictive performance for unseen plots, years, or regions. The study also did not evaluate fertiliser recommendations or their agronomic benefits. The operational costs of soil sampling, laboratory analysis, and repeated UAV flights with calibration, as well as the feasibility of routine use by farmers, remain to be evaluated. Further validation using held-out plots, data from multiple years and sites, and field trials is needed before this approach can be considered for practical nitrogen management.

### 3.3. Performance Differences and Applicability Analysis of Machine Learning Models

The overall STN estimation performance of the four machine learning models was XGBoost > GBDT > RFR > KNN, with average validation set R^2^ values of 0.796, 0.703, 0.691, and 0.673, respectively, under feature fusion. Multi-source fusion increases the complexity of feature dimensions and interactions, thereby allowing tree-based ensemble models—which are better suited to handling non-linear relationships—to demonstrate their advantages [29]. Previous studies have shown that non-linear machine learning models outperform traditional linear methods in the inversion of summer maize canopy parameters [30].

Performance differences between models are related to their learning mechanisms and feature structures. RFR enhances the model’s tolerance to noise and outliers through Bootstrap sampling and random feature selection, and its suitability for nitrogen remote sensing estimation has been supported by existing research [31]. However, as RFR involves training each decision tree independently and then averaging the results, it lacks stepwise error correction and is less capable of capturing complex feature interactions than Boosting models. Both GBDT and XGBoost reduce prediction bias through iteration; however, XGBoost achieves an average R^2^ approximately 13.2 per cent higher than GBDT when features are fused. Compared with GBDT, XGBoost further utilises second-order gradient information and controls model complexity through regularisation and feature sampling, which helps to improve generalisation ability [32]. Under conditions where sample sizes are consistent across different reproductive stages and there are a large number of combined feature variables, these mechanisms help to improve the model’s adaptability to both the samples and the combined features. However, as KNN relies on the distance between samples for prediction, its ability to distinguish distances is somewhat compromised as the number of combined features increases; consequently, its accuracy gains are lower than those of tree-based ensemble models.

In terms of model fit, there are certain differences in how well different algorithms adapt to the feature structure in this study, and similar patterns of model fit have also been observed in previous research. A study on the UAV multi-spectral inversion of total nitrogen (TN) in the rhizosphere soil of silage maize found that PLSR, following feature selection, exhibited good predictive performance [33], indicating that linear models still demonstrate good adaptability when feature variables are effectively selected and the relationships between variables are relatively clear. This study integrated multi-source features including Band, VIs, and TF, resulting in more complex non-linear interactions between variables; consequently, XGBoost demonstrated superior fitting capability. XGBoost’s observed advantage may partly depend on patterns specific to the experimental field, and its predictive stability under independent conditions requires further validation. Previous studies have shown that XGBoost achieves good predictive results in the hyperspectral inversion of total nitrogen in winter wheat [32] and outperforms RF and SVM in the hyperspectral estimation of soil organic matter [34], further demonstrating its good adaptability in handling the non-linear relationships between complex spectral features and soil properties.

In this study, STN measurement refers to laboratory determination using the Kjeldahl method, whereas STN estimation refers to indirect prediction from UAV-derived canopy features. The models describe statistical relationships between canopy characteristics and laboratory-measured STN without explicitly representing radiative transfer or soil biogeochemical processes, limiting mechanistic interpretation of water stress and microtopographic effects. The UAV-derived canopy features may reflect both STN variation and the combined effects of irrigation and nitrogen application. With only one plot per treatment, these influences could not be separated. Therefore, the model’s predictive relationships cannot be attributed exclusively to STN, and further validation using independently replicated plots is needed. Furthermore, this study covered only the 2025 growing season (May–October), and the effects of interannual variations in precipitation and temperature could not be assessed. These factors may influence nitrogen dynamics and canopy spectral responses. The findings should therefore be interpreted within the conditions of this experimental site; their transferability to different soil types, climates, maize cultivars and management practices requires independent validation across multiple years and sites. In summary, the second-order gradient optimisation, regularisation, and feature sampling mechanisms of XGBoost are well-suited to the multi-source fusion of features and complex non-linear relationships in this study, thereby achieving a high level of accuracy in STN estimation.

## 4. Materials and Methods

### 4.1. Overview of the Study Area

**Figure 13:**
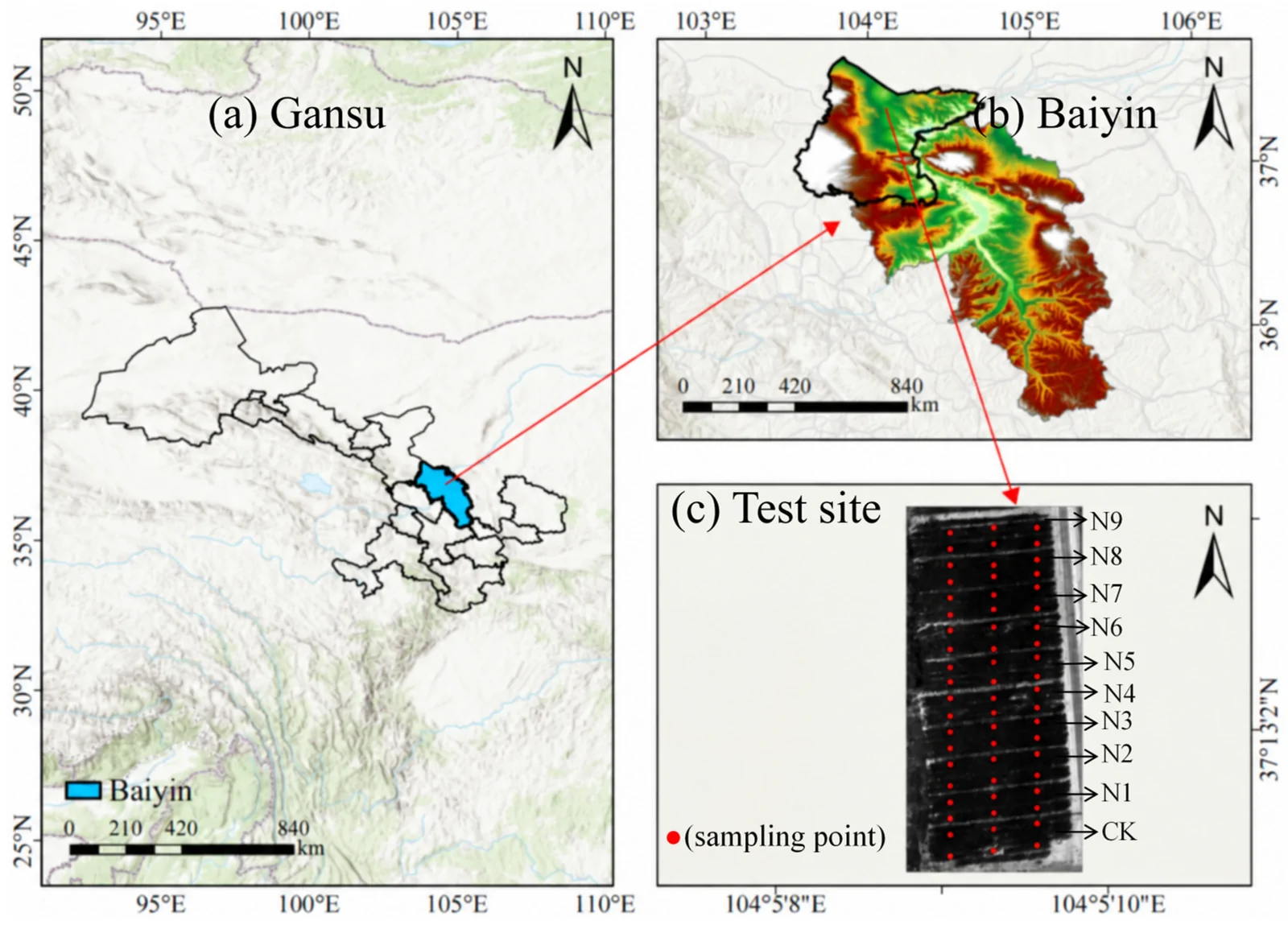
Overview of the study area. Note: (**a**) location of Baiyin City, highlighted in cyan; (**b**) terrain of Baiyin City, with different colors representing variations in elevation; (**c**) experimental site and sampling point distribution in Jingtai County. Red dots indicate sampling points, and red arrows link the corresponding locations across panels.

The experiment was conducted between May and October 2025 at the Jingtai-Chuan Electric Power-Driven Irrigation Water Resources Utilisation Centre Irrigation Experimental Station in Baiyin City, Gansu Province (103°20′–104°04′ E, 37°26′–38°41′ N, elevation 1630.5 m; Figure 13) [20]. The region has a continental temperate arid climate, with a long-term average precipitation of 190.9 mm. Precipitation is unevenly distributed, concentrated between July and September, accounting for more than 63 per cent of the annual total. The average annual evaporation is 2289.9 mm, the frost-free period is 190 days, and the average annual sunshine duration is 2726.2 h. The annual mean temperature is 8.8 °C, with maximum temperatures reaching 35.0 °C, and accumulated temperature ≥ 10 °C totalling 3038.4 °C.

### 4.2. Experimental Design

Ten irrigation–nitrogen treatments were established. Under drip irrigation, treatments N1, N2, and N3 received 300, 225, and 150 kg N·ha^−1^, respectively. Under furrow irrigation, treatments N4, N5, and N6 received the same three nitrogen application rates, respectively. Under seepage irrigation, treatments N7, N8, and N9 also received these three rates, respectively. The control treatment (CK) received traditional flood irrigation and 225 kg N·ha^−1^. Each treatment was represented by one experimental plot, giving ten plots in total. Six fixed sampling points were established within each plot as subsamples. No independent plot replication was included within each treatment.

The maize cultivar used in this experiment was Kenyu 50. Maize was sown on 5 May 2025 and harvested on 1 October 2025, with a growing season of approximately 150 days.

Each plot measured 110.5 m^2^ (22.1 m × 5.0 m), with a spacing of 1 m between plots and two buffer rows. The maize was planted with a row spacing of 60 cm and a plant spacing of 25 cm, at a machine-sown depth of 3–5 cm, resulting in a planting density of approximately 6.6 × 10^4^ plants·ha^−1^.

Urea (46% nitrogen content) was used as the nitrogen source. The nitrogen application rates for treatments N1, N2, and N3 were 300, 225, and 150 kg N·ha^−1^, respectively, corresponding to urea application rates of 652.2, 489.1, and 326.1 kg·ha^−1^. These nitrogen application rates refer to nitrogen supplied by urea alone and do not include nitrogen contributed by diammonium phosphate. Urea was applied in instalments at a ratio of 30% as base fertiliser, 40% at the jointing stage, and 30% at the tasselling stage. Diammonium phosphate was used as the phosphorus fertiliser at 1.03 kg per plot (93.21 kg·ha^−1^), and potassium sulphate was used as the potassium fertiliser at 0.82 kg per plot (74.21 kg·ha^−1^), based on a plot area of 110.5 m^2^. These phosphorus and potassium fertiliser application rates refer to fertiliser product mass. The application rates and timing of phosphorus and potassium fertilisers were consistent across all treatments. Other field management practices were also consistent across treatments [22,35].

### 4.3. Monitoring Procedure for STN Content

**Figure 14:**
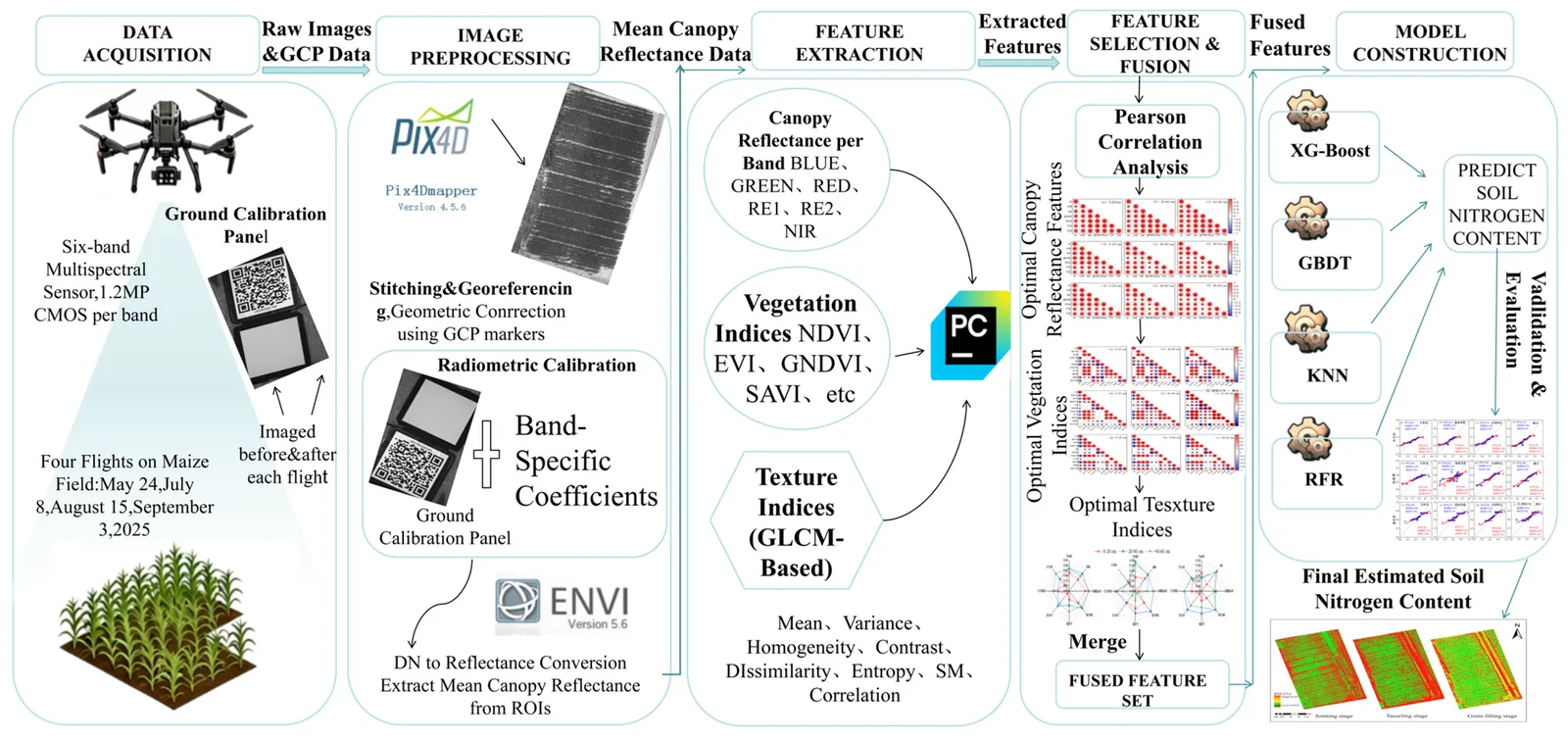
Workflow for estimating soil total nitrogen (STN) in maize using UAV multi-spectral data.

This study utilised a drone equipped with a multi-spectral sensor to acquire multi-spectral imagery of maize at three key growth stages (jointing stage, tasselling stage, and grain filling stage). The multi-spectral images were pre-processed to extract canopy reflectance, vegetation indices, and texture indices. Pearson correlation analysis was used to screen for feature variables significantly correlated with STN, and both single-feature and fused feature sets were constructed. Models for estimating maize soil nitrogen content were developed using XGBoost, GBDT, KNN, and RFR; the specific workflow is shown in Figure 14.

### 4.4. Data Acquisition and Processing

#### 4.4.1. Determination of STN Content

Six fixed soil sampling points were established in each of the ten experimental plots, giving sixty sampling points in total. These points were treated as within-plot subsamples rather than independent experimental replicates. At each growth stage, soil samples were collected from three soil layers—0–20 cm, 20–40 cm, and 40–60 cm—at each sampling point. Consequently, a total of 180 soil samples were obtained for each growth stage, with 60 samples per soil layer, and these were labelled with the sample code ‘Stage—Plot—sampling unit—soil layer’. Approximately 1 kg of soil was retained using the quartering method; after removing gravel and plant debris, the samples were air-dried, ground, and sieved through a 0.149 mm nylon sieve. Approximately 1 g of the prepared soil sample was weighed and digested with 10 mL of sulphuric acid and 3 g of catalyst. The soil total nitrogen content was then determined using a Kjeldahl analyser (Kjeltec 8400, FOSS) [25,33]. Three parallel laboratory determinations were carried out for each soil sample, and the mean value was calculated.

Soil samples were collected on 8 July, 15 August, and 3 September 2025, corresponding to the jointing, tasselling, and grain filling stages, respectively. At each growth stage, soil sampling and UAV image acquisition were conducted on the same day.

#### 4.4.2. Remote Sensing Data Acquisition

Under clear, windless conditions with ample sunlight, multi-spectral imagery was acquired using a DJI Matrice 300 RTK quadcopter (DJI Innovation, Shenzhen, China) equipped with an MS600 Pro multi-spectral camera (YUSENSE Information Technology and Equipment (Qingdao) Co., Ltd., Qingdao, China). The multi-spectral system comprises six independent 1.2 MP CMOS sensors covering the Blue, Green, Red, Rededge 1, Rededge 2, and near-infrared (NIR) bands. The imagery was processed using Pix4DMapper and radiometrically calibrated using diffuse reflectance panels and the reflectance coefficients of each band to derive canopy reflectance (Table 2). The flight altitude was 30 m, with forward and side-to-side overlap rates of 80% and 70%, respectively. The flight speed was 2.7 m·s^−1^, and operations took place between 12:00 and 13:00, with the camera pointing vertically downwards whilst flying along a pre-set flight path. Images of ground calibration panels were acquired before and after each flight (ground sampling distance of 2.16 cm) [36] to correct for changes in illumination and ensure data consistency.

#### 4.4.3. Pre-Processing of Remote Sensing Images

In this study, four ground control points (GCPs) were established in the study area, with their spatial coordinates acquired using Real-Time Kinematic (RTK) positioning technology. Image stitching and georeferencing were carried out using Pix4D Mapper 4.8.0 software, and geometric fine-tuning was performed by manually labelling the GCPs. Following this processing, the generated digital orthophotos exhibited root mean square errors (RMSE) of 0.22 m, 0.31 m, and 0.12 m in the x, y, and z directions, respectively. Radiometric correction was performed using a standard white reference panel to eliminate the influence of varying lighting conditions on the spectral information of the canopy. Following the completion of geometric and radiometric corrections, the digital number (DN) values of the image pixels were converted to reflectance using ENVI 5.6 software. The average reflectance spectra of the maize canopy were then extracted from the regions of interest (ROIs) corresponding to each sampling point, ultimately yielding reflectance data for each band for further analysis.

### 4.5. Feature Extraction

#### 4.5.1. Calculation of Vegetation Indices

The multi-spectral bands used in this study were Blue (B), Green (G), Red (R), Rededge 1 (RE1), Rededge 2 (RE2), and near-infrared (NIR). A 1 m × 1 m square region of interest (ROI) was defined for each soil sampling point. At each growth stage, the mean reflectance of each band was extracted within the corresponding ROI. The ten vegetation indices (VIs) listed in Table 3 were then calculated from these ROI-averaged band reflectances using the corresponding formulas.

#### 4.5.2. Texture Feature Extraction

This study utilised Green-band reflectance imagery as its basis and employed the Grey-Level Co-occurrence Matrix (GLCM) in ENVI 5.6 to extract textural features. The image was quantised into 64 grey levels, with a 3 × 3-pixel sliding window and a pixel offset of 1; texture values were calculated for the four directions of 0°, 45°, 90°, and 135°, and the directional average was taken. Candidate indicators included mean (MEAN), variance (VAR), homogeneity (HOM), dissimilarity (DISS), contrast (CON), entropy (ENT), second-order moment (SM), and correlation (CORR) [24,25,26,46]. The mean values for each texture raster were also extracted using a fixed sampling unit ROI and matched with the corresponding soil samples.

### 4.6. Pearson Correlation Analysis

Pearson correlation analysis was used to screen predictors associated with STN [47]. However, screening based solely on correlations with STN may overlook non-linear relationships and interactions and does not directly assess redundancy among predictors. In particular, VIs are derived from spectral bands, so Band and VIs may contain overlapping information when combined in the fused input. The Pearson correlation coefficient ranges from −1 to 1. A higher absolute value of r indicates a stronger linear correlation between the predictor variable and the target variable. According to the evaluation criteria for the Pearson correlation coefficient (|r| ≥ 0.8 indicates a high correlation between the two variables; 0.5 ≤ |r| < 0.8 indicates a moderate correlation; 0.2 ≤ |r| < 0.5 indicates a low correlation; |r| < 0.2 indicates that the two variables are essentially uncorrelated), correlation analyses were conducted on spectral reflectance, vegetation indices, and textural features, using the following formulae:

(1) $$r=\frac{\sum \left(X_{i}-\bar{X}\right)\left(Y_{i}-\bar{Y}\right)}{\sqrt{\sum \left(X_{i}-\bar{X}\right)\cdot }\sqrt{\sum \left(Y_{i}-\bar{Y}\right)}}$$

In the equation, r represents the Pearson correlation coefficient; X_i_ and Y_i_ are the observed values of the two variables; and $\bar{X}$ and $\bar{Y}$ are the sample means of the variables.

### 4.7. Model Construction and Accuracy Evaluation

Model hyperparameters were optimised using grid search and four-fold cross-validation within the training set. The parameter combination yielding the highest mean R^2^ across the four validation folds was selected. This study utilised RFR, GBDT, KNN, and XGBoost to construct STN estimation models, with the aim of comparing the ability of different algorithms to fit the non-linear relationship between STN and multi-source remote sensing features [5,28,29,34,48,49]. The six spectral bands, ten VIs, and eight TF were candidate predictors. Following screening, each individual feature input contained three predictors, while the fused input contained nine. Each model was trained using 42 samples and validated using 18 samples for each growth stage–soil depth combination. For RFR, the number of decision trees was set to 300, the maximum depth to 6, the minimum number of samples per leaf node to 1, and the maximum number of features was determined using the square root method [28,48]. For GBDT, the number of decision trees was set to 300, the learning rate to 0.05, the maximum depth to 3, and the subsample ratio to 0.8 [49]. For KNN, the number of neighbours K was set to 5, with predictions based on Euclidean distance and distance weighting; input variables were standardised according to the mean and standard deviation of the training set, and the same transformation was applied to the validation set [19]. For XGBoost, the number of boosted trees was set to 300, the maximum depth to 3, the learning rate to 0.05, the row and column sampling ratios to 0.8 each, and the L2 regularisation coefficient to 1 [5,29,34].

Model accuracy was evaluated using the coefficient of determination (R^2^), root mean square error (RMSE), and mean absolute error (MAE). The closer R^2^ is to 1, the stronger the model’s ability to explain STN variation; RMSE reflects the overall deviation between predicted and observed values, with a smaller value indicating lower prediction error [50]; MAE is the average of the absolute errors between predicted and observed values, and directly reflects the model’s average prediction error; a smaller value indicates better predictive performance [11].

### 4.8. Data Processing

In this study, Microsoft Excel 2019 was used for data organisation; Pix4D Mapper 4.8.0 and ENVI 5.6 were utilised to pre-process UAV multi-spectral imagery, extracting average canopy reflectance and texture features, and subsequently calculating vegetation indices (VIs) and texture features (TF); ArcGIS Pro 3.1.6 was used to extract datasets for each sample point based on the vector boundaries of the experimental plots; correlation analysis, feature selection, model building, and accuracy assessment were carried out within the PyCharm 2025.2.6.1 environment; Origin 2021 was used for graphing.

## 5. Conclusions

(1) The prediction accuracy of STN decreased with increasing soil depth, with the 0–20 cm soil layer showing the highest accuracy across all growth stages. The overall performance among growth stages followed the order of grain filling stage > tasseling stage > jointing stage, with validation set R^2^ values of 0.761–0.833 for XGBoost using feature fusion.
(2) Feature fusion improved STN estimation accuracy compared with individual Band, VIs, or TF inputs, and XGBoost exhibited the best overall performance among the evaluated models. With the integration of Band+VIs+TF, the validation set R^2^ values of XGBoost in the 0–20 cm soil layer ranged from 0.761 to 0.833 across different growth stages, with the highest accuracy achieved at the grain filling stage.
(3) The four machine learning models exhibited distinct differences in STN estimation performance, following the order of XGBoost > GBDT > RFR > KNN. Under the random training–validation split, XGBoost with Band+VIs+TF achieved the highest observed prediction accuracy in this dataset. Its predictive performance on independent plots, years, and sites, as well as its practical value for fertiliser management, remains to be validated.

## Figures and Tables

**Table 1:** Statistical characteristics of STN content.

Growth Stage	Soil Depth/cm	Sample Size	MAX/ g·kg^−1^	MIN/ g·kg^−1^	MEAN/ g·kg^−1^	SD/g·kg^−1^	CV/%
Jointing Stage	0–20	60	0.794	0.510	0.669	0.067	10.51
	20–40	60	0.716	0.331	0.506	0.109	21.64
	40–60	60	0.807	0.300	0.569	0.143	25.26
Tasseling Stage	0–20	60	0.753	0.334	0.567	0.136	24.10
	20–40	60	0.895	0.371	0.578	0.106	18.35
	40–60	60	0.568	0.279	0.435	0.067	15.43
Grain Filling Stage	0–20	60	0.980	0.666	0.834	0.055	6.65
	20–40	60	0.935	0.521	0.780	0.091	11.72
	40–60	60	0.994	0.383	0.682	0.106	15.56

**Table 2:** Spectral Bands, Centre Wavelengths, Band Widths, and Reflectances.

Band	Centre Wavelength/nm	Band Width/nm	Reflectance%
Blue	450	35	60
Green	555	25	60
Red	660	20	60
Rededge 1	720	10	60
Rededge 2	750	15	60
NIR	840	35	60

**Table 3:** Summary of vegetation indices.

VIs	Formula	References
NDVI	(NIR − R)/(NIR + R)	[37]
RVI	NIR/R	[38]
GNDVI	(NIR − G)/(NIR + G)	[39]
GI	G/R	[40]
RGRI	R/G	[41]
OSAVI	1.16(NIR − R)/(NIR + R + 0.16)	[42]
DVI	NIR–R	[43]
MSR	(NIR/R − 1)/√*NIR*/*R* + 1	[44]
SRPI	B/R	[45]
NDVI	(G − B)/(G + B)	[45]

Note: B, G, R, RE1, RE2, and NIR represent the spectral reflectance at 450, 555, 660, 720, 750, and 840 nm, respectively, obtained by the MS600 Pro multi-spectral camera.

## Data Availability

The data supporting the findings of this study, including soil total nitrogen measurements, UAV multi-spectral imagery-derived spectral reflectance, vegetation indices, texture features, and datasets used for machine learning model development and validation, are not publicly available due to privacy restrictions but may be obtained from the corresponding author upon reasonable request.
